# One-Pot Synthesis and Characterization of Novel Shape-Memory Poly(ε-Caprolactone) Based Polyurethane-Epoxy Co-networks with Diels–Alder Couplings

**DOI:** 10.3390/polym10050504

**Published:** 2018-05-06

**Authors:** Katalin Czifrák, Csilla Lakatos, József Karger-Kocsis, Lajos Daróczi, Miklós Zsuga, Sándor Kéki

**Affiliations:** 1 Department of Applied Chemistry, University of Debrecen, Egyetem tér 1, H-4032 Debrecen, Hungary; czifrak.katalin@science.unideb.hu (K.C.); lakatoscsilla@science.unideb.hu (C.L.); zsuga.miklos@science.unideb.hu (M.Z.); 2 Department of Polymer Engineering, Faculty of Mechanical Engineering, Budapest University of Technology and Economics, Műegyetem rkp. 3, H-1111 Budapest, Hungary; 3 MTA–BME Research Group for Composite Science and Technology, Műegyetem rkp. 3, H-1111 Budapest, Hungary; 4 Department of Solid State Physics, University of Debrecen, Bem tér 18/b, H-4026 Debrecen, Hungary; lajos.daroczi@science.unideb.hu

**Keywords:** epoxy resin, polyester polyurethane, Diels–Alder adduct, polycaprolactone, co-network, shape recovery, stress–strain behavior, modeling

## Abstract

The present work aimed at the preparation and investigation of different epoxy-polyurethane (EP-PU) co-networks. The EP-PU co-networks were obtained by applying two different synthetic strategies, in which the coupling element, the Diels–Alder (DA) adduct, was prepared previously or formed “in situ” in the reaction between furan functionalized polyurethane and furfuryl amine-diglycidyl ether bisphenol-A oligomers (FA_DGEBA). For the synthesis of these EP-PU networks, poly(ε-caprolactone)-diol (PCD, *M*_n_ = 2 kg/mol) and poly(ε-caprolactone) (PCL) with different molecular weights (*M*_n_ = 10, 25 and 50 kg/mol) and 1,6-hexamethylenediisocyanate (HDI) were used. The EP-PU co-networks were characterized by Attenuated Total Reflectance Fourier-Transform Infrared spectroscopy (AT-FT-IR), differential scanning calorimetry (DSC) and dynamical mechanical analysis (DMA). Scanning electron microscopy (SEM) was applied to assess the morphology of the EP-PU samples. It was demonstrated that the stress–strain curves for the EP-PUs could be interpreted based on the Standard Linear Solid (SLS) model. The DMA traces of some EP-PUs (depending on the composition and the synthetic method) revealed a plateau-like region above the melting temperature (*T*_m_) of PCL confirming the presence of cross-linked structure. This feature predicted shape memory (SM) behavior for these EP-PU samples. Indeed, very good shape fixity and moderate shape recovery were obtained. The shape recovery processes of these EP-PU samples were described using double exponential decay functions.

## 1. Introduction

Epoxy resins (EPs) are well-known for their versatile applications covering coatings, adhesives and matrices of composites [1,2,3,4]. The widespread use EPs is mostly due to their good mechanical properties, and high thermal and chemical stabilities. However, their cross-linked networks make EPs intrinsically brittle materials. Thus, EPs exhibit low toughness and ductility which strongly limit their application fields [5]. To overcome these limitations, one of the strategies is to introduce flexible polymer chains into the network providing it with better deformability and yielding higher toughness. For the network modification polyurethanes (PUs) are often the preferred additives due their versatile chemistry [6].

There are numerous reports for the preparation EP-PU systems in which different interpenetrating network (IPN) were formed [7,8,9,10,11,12]. Furthermore, incorporations of Diels–Alder (DA) adducts into different polymeric systems have paved the way for the preparation of networks with thermally reversible couplings [13,14,15,16]. The related systems exhibit self-healing [17,18,19], shape memory (SM) behavior [20,21], and even a combination of self-healing and SM, denoted as Diels–Alder shape memory-assisted self-healing [22].

Stemming from previous studies, it can be expected that the relative amount of DA adducts strongly affect the abovementioned functional properties. It is intuitive that reduction of the chain segment’s length between DA moieties is a straightforward approach to enhance the concentration of DA couplings. This is frequently followed in poly(ε-caprolactone) (PCL)-containing polymers [19,20], covering also PUs [21,22]. Note that, in these systems, the melting/crystallization of PCL serves for the reversible switch function of their SM behavior. There is a further, yet less explored, possibility following the same principle (i.e., segment’s length reduction), namely creation of co-networks involving DA linkages. For the generation of co-networks, the combination of EP and PU chemistries seem to be a proper way. This is reasoned by the chemical reactions between them along with the fact that the most used polymers to demonstrate the possibilities of thermoreversible DA couplings are EP- and PU-based ones. Thus, in this study, we focused on the synthesis and characterizations of EP-PU co-networks containing both crystalline PCL segments and DA adducts to achieve good SM properties. In this work, two synthesis methods were followed. In the first approach, the co-networks were derived via the reaction of isocyanate terminated poly(ε-caprolactone) prepolymers with the hydroxyl terminated Diels–Alder adduct (synthesized by the reaction of furfuryl alcohol with 1,1′-(methylene di-4,1-phenylene)bismaleimide (BMI) and bisphenol A diglycidyl ether oligomer (DGEBA). According to the second synthetic path, DGEBA was first reacted with furfurylamine (FA), and this compound (FA_DGEBA oligomer) reacted further with furanyl terminated poly(ε-caprolactone) containing PU in the presence of BMI. The DA adducts were in both cases reaction products of furan and maleimide, however, in the first case they were previously synthesized, whereas in the second pathway in situ produced. The effects of the composition and the DA-adducts on the mechanical, thermomechanical properties and morphologies of these crosslinked EP-PU hybrids were assessed and discussed.

## 2. Materials and Methods

### 2.1. Materials

For the synthesis of the EP-PU co-networks, the following chemicals were used: Poly(ε-caprolactone)-diol (PCD, *M*_n_ = 2 kg/mol) and poly(ε-caprolactone) (PCL, *M*_n_ = 10 kg/mol) from Sigma-Aldrich (Darmstadt, Germany). PCLs with *M*_n_ = 25 and 50 kg/mol were purchased from Perstorp Holding AB (Malmö, Sweden), 1,6-hexamethylene diisocyanate (HDI) (reagent grade), Furfurylamine (FA), Furfurylalcohol (Falc), 1,1′-(methylene di-4,1-phenylene)bismaleimide (BMI) (reagent grade), *N,N*-dimethylformamide (anhydrous 99.8%) and Tin(II)ethylhexanoate (reagent grade) were obtained from Sigma-Aldrich (Darmstadt, Germany). Bisphenol A diglycidyl ether type EP oligomer (DGEBA, epoxy equivalent weight: 182–192 g/mol) was provided by Alvinplast (Budapest, Hungary). For crosslinking, Jeffamine EDR-176 (amine equivalent weight: 44 g/mol) from Hunstman Corporation (Pétfürdő, Hungary) were applied. Toluene (analytical grade) from Sigma-Aldrich (Darmstadt, Germany) was distilled over P_2_O_5_ and stored on sodium wire until use.

### 2.2. Synthesis of DA Adduct from Furfuryl Alcohol and BMI


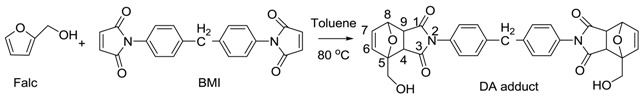


The synthesis of the DA adduct –see the above scheme- was performed similarly to those reported in Ref. [23]. Furfuryl alcohol (Falc) (1 g) (880 µL, 1.02 × 10^−2^ mol, 2 equivalent) and BMI (1.826 g, 5.095 × 10^−3^ mol, 1 equivalent) were dissolved in dry toluene (10 mL) and heated for 2 days at 75–80 °C. The precipitate was filtrated and washed with diethyl ether to give 2.60 g 92% yellow crystalline product, mainly the exo isomer. ^1^H NMR: (360 MHz, CDCl_3_) δ (ppm): (exo isomer) 7.40–7.23 (8H, m, ArH), 6.74 (2H, d, *J* = 5.6 Hz, H-6, H-6′), 6.66 (2H, d, *J* = 5.6 Hz, H-7, H-7′), 5.45 (2H, d, *J* < 1Hz, H-8, H-8′), 4.23 (2H, s, OH), 4.11 (2H, s, ArCH_2_Ar), 3.23 (2H, dd, *J* = 6.5, <1 Hz, -CH_2_OH), 3.19 (2H, dd, *J* = 6.5, <1 Hz, -CH_2_OH), 2.45–2.43 (4H, m, H-9, H-9′, H-4, H-4′).

### 2.3. Functionalization of DGEBA Resin

**Figure 1:**
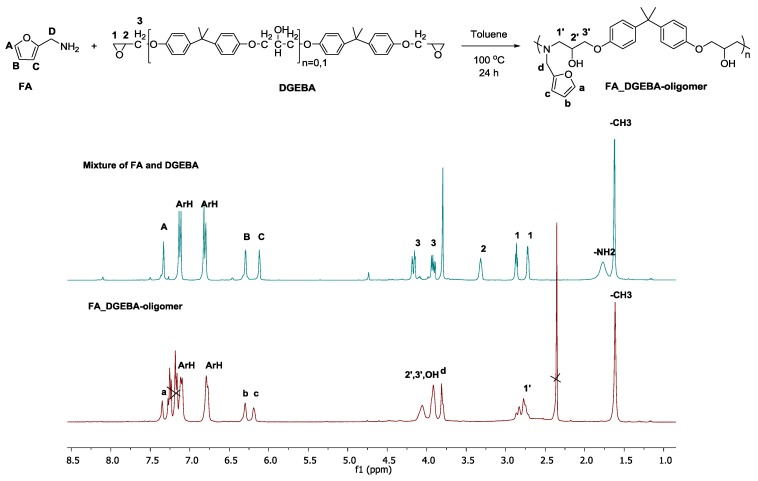
^1^H NMR spectra of FA_DGEBA oligomer and its starting compounds as well as their assignations.

**Figure 2:**
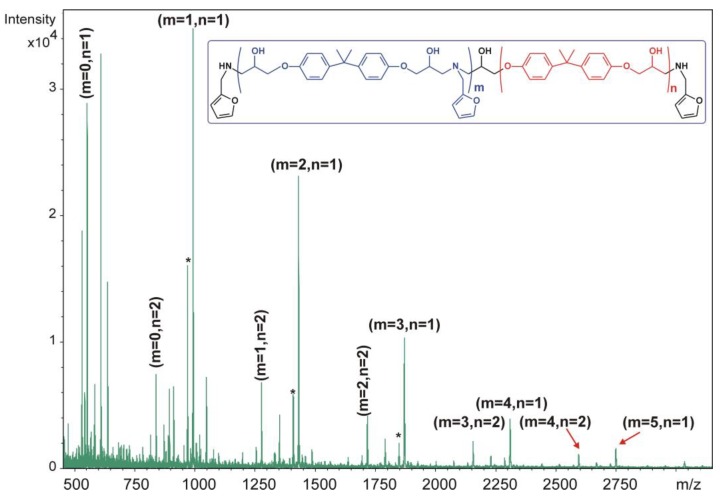
MALDI-TOF MS spectrum of FA_DGEBA oligomer. The numbers at the top of the peaks stand for the number of repeat units. Peaks denoted by (*) are the protonated oligomers ([M + H]^+^).

The synthesis route was similar to that one reported in Ref. [24]. FA (2.45 mL, 2.8 × 10^−2^ mol, 2 equivalent) and DGEBA (5 g, 1.4 × 10^−2^ mol, 1 equivalent) were mixed in dry toluene (25 mL) and reacted at 100 °C for 24 h. The obtained dark red solution was used in further reactions after ^1^H NMR and MALDI-TOF MS (the experimental conditions detailed later) analysis (see Figure 1 and Figure 2).

As seen in Figure 1, the signal at 3.32 ppm, corresponding to that of the epoxy protons of DGEBA, completely disappeared indicating complete reaction of the epoxy groups with the amine moiety of FA. In addition, MALDI-TOF MS measurements confirmed the presence of FA_DGEBA oligomers with different number of repeat units (m, n) and FA end-groups as seen in Figure 2.

The *M*_n_ and the number average functionality of the FA_DGEBA oligomer (*f*_n_) were determined by MALDI-TOF MS by using Equations (1) and (2):

(1) $$M_{n}=\frac{\sum_{i=1}^{k}I_{i}M_{i}}{\sum_{i=1}^{k}I_{i}}$$

(2) $$f_{n}=\frac{\sum_{i=1}^{k}I_{i}f_{i}}{\sum_{i=1}^{k}I_{i}}$$

where *M*_i_, *f*_i_ and *I*_i_ are the mass (without cation), the functionality and the MALDI-TOF MS intensity of the *i*th member of the FA_DGEBA oligomer series, respectively. 

The values of *M*_n_ and *f*_n_ of the FA_DGEBA oligomer series were determined to be 1150 g/mol and 3.3 g/mol, respectively. In these calculations (Equations (1) and (2)), it was assumed that the ionization efficiencies are independent of the mass of the oligomer species.

### 2.4. Synthesis of DGEBA-PU Co-network with DA Adduct

**Scheme 1:**
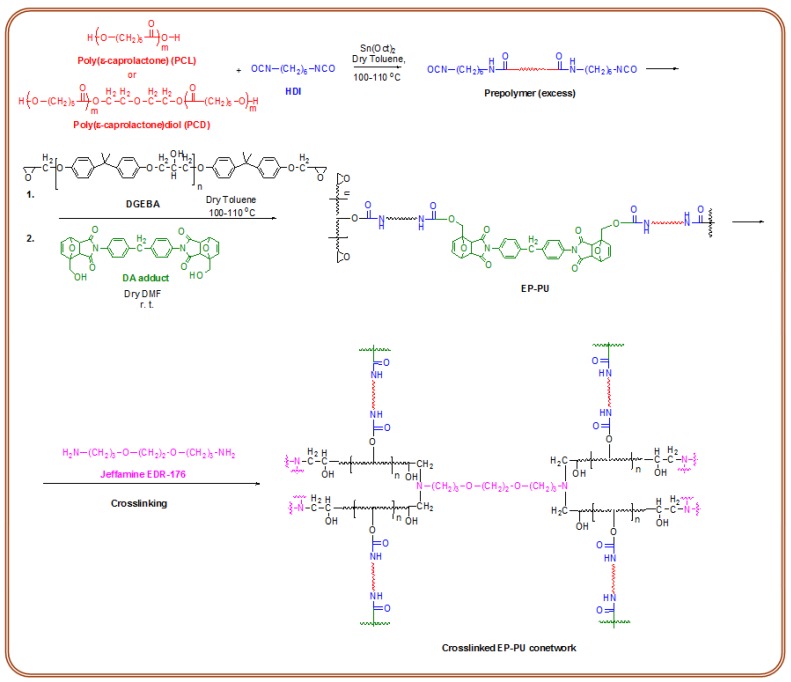
Synthetic pathway for the preparation of EP-PU co-networks (EP-PUs 1-15) containing DA-adducts.

The DGEBA-PU co-networks contain PCLs with different molecular weights (i.e., PCD and PCL), EP and DA adduct, also in different amounts. The synthetic pathway of the preparation of EP-PU 1-15 samples is summarized in Scheme 1. Samples EP-PU 1–15 were prepared as described in the following section.

**Representative procedure for the synthesis of EP-PU 2:** 0.6 g PCD (3 × 10^−4^ mol, *M*_n_ = 2 kg/mol) was dissolved in hot toluene 60–80 °C (50 mL) under a nitrogen atmosphere in a 250 mL four-neck flask (equipped with mechanical stirrer, dropping funnel, condenser and nitrogen inlet). Tin(II) ethylhexanoate in 2 mol % amount was used as catalyst. To obtain the prepolymer with isocyanate end groups, 0.10 g (97 μL) HDI (6 × 10^−4^ mol, 2 equivalent) was added to the mixture and reacted for 2 h at 100–110 °C. Then, the epoxy resin (DGEBA) 0.11 g (3 × 10^−4^ mol, 1 equivalent) was added in one portion and stirred further 1 h at 100–110 °C. The DA adduct 0.165 g (3 × 10^−4^ mol, 1 equivalent) and were dissolved in dry DMF (1 mL) and added to the isocyanate ended prepolymer solution in one portion at room temperature and the reaction mixture was kept at this temperature for 24 h. The composition of this EP-PU 2 is PCD(2)-HDI-DA adduct-DGEBA/1:2:1:1, where (2) refers to the *M*_n_ of PCD expressed in kg/mol unit. EP-PUs 1 and 3–9 were synthesized according to the procedure described above (EP-PU networks). 

To crosslink EP-Pus, Jeffamine (JA) as curing agent in 1 equivalent to DGEBA was added to the reaction mixture at 40 °C and stirred for 3 h vigorously (crosslinked EP-PU co-networks, designated as EP-PU 10–15). The corresponding molar ratios in the feed of the synthesized EP-PU 1–15 are summarized in Table 1.

The mixtures were poured onto Teflon^®^ plates and dried in air yielding the final EP-PU co-networks in form of elastic films. The final co-networks were obtained after 24 h curing at 60 °C.

### 2.5. Synthesis of FA-DGEBA Oligomer-PU Networks

**Scheme 2:**
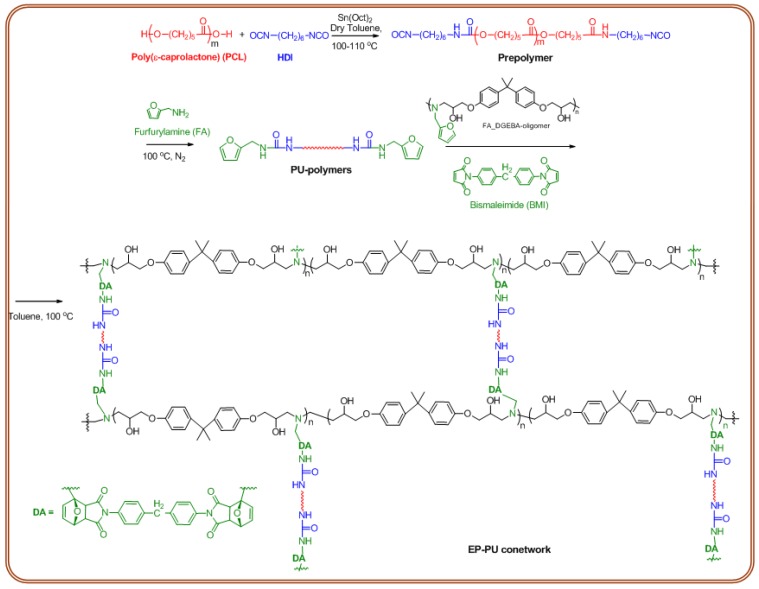
Synthetic pathway for the preparation of samples EP-PU 16–24 containing DA-adducts.

The synthesized EP-PU co-networks differed in the *M_n_* of PCL and weight ratio of the FA_DGEBA oligomer applied. The synthetic approach for the preparation of EP-PU 16–24 samples is summarized in Scheme 2. EP-PU 16 sample was prepared as described below.

**Representative procedure for the synthesis of EP-PU 16:** 3.0 g PCL (3 × 10^−4^ mol, *M*_n_ = 10 kg/mol) was dissolved in hot toluene 60–80 °C (50 mL) under a nitrogen atmosphere in a 250 mL four-neck flask (equipped with mechanical stirrer, dropping funnel, condenser and nitrogen inlet). Tin(II) ethylhexanoate in 2 mol % amount was used as catalyst. To prepare the prepolymer with isocyanate end-groups, 0.10 g (97 μL) HDI (6 × 10^−4^ mol, 2 equivalent) was added to the reaction mixture and reacted for 2 h at 100–110 °C. To form the DA adduct, 0.06 g (53 μL) FA (6 × 10^−4^ mol, 2 equivalent) was added to the isocyanate-capped prepolymer solution in one portion at 100 °C and kept there for 1 h. Afterwards, FA_DGEBA oligomer in toluene solution (0.34 g, 3 × 10^−4^ mol) and 0.11 g (3 × 10^−4^ mol) BMI were added to the reaction mixture and stirred at 100–110 °C for 1 h. Accordingly, the composition of this EP-PU 16 sample is PCL(10)-HDI-FA-FA_DGEBA oligomer-BMI/1:2:2:1:1, where (10) refers to the *M*_n_ of PCL expressed in kg/mol unit. EP-PUs 17–24 were synthesized according to the procedure described above. The corresponding molar ratios in the feed of the EP-PUs 16–24 synthesized are given in Table 2.

The mixtures containing the final EP-PU co-networks were poured onto Teflon^®^ plates and dried in air resulting in elastic films.

### 2.6. Characterization

^1^H NMR spectra were recorded with a Bruker AM 360 (360/90 MHz for ^1^H/^13^C) spectrometer (Karlsruhe, Germany). Deuterated chloroform was used as solvent. Chemical shifts were referenced to the ^1^H signal of Me_4_Si, which was used as standard.

For the Matrix-Assisted Laser Desorption/Ionization Time-of-Flight Mass Spectrometry (MALDI-TOF MS) measurements a Bruker BIFLEX III^TM^ mass spectrometer equipped with a time-of-flight (TOF) mass analyzer (Bremen, Germany) was used. In all cases, 19 kV acceleration voltage was applied with pulsed ion extraction (PIE^TM^). The ions were detected in the reflectron mode (20 kV). A nitrogen laser (337 nm) operating at 4 Hz was used to produce laser desorption and 100 shots were summed. The MALDI-TOF MS spectra were externally calibrated with poly(ethylene glycol) standard (*M*_n_ = 1540 g/mol).

Samples for MALDI-TOF MS were prepared with 2,5-dihydroxy benzoic acid (DHB) matrix. The matrix was dissolved in tetrahydrofuran (THF) at a concentration of 20 mg/mL. The matrix solution, the FA_DGEBA oligomer mixture (10 μL reaction mixture dissolved in 90 μL THF and sodium trifluoroacetate solution (5 mg/mL in THF) used as the cationization agent to promote the ionization) were mixed in a 10:2:1 (*v*/*v*) ratio (matrix/analyte/cationization agent). A volume of 0.5 μL of the solution was deposited onto a metal sample plate and allowed to air-dry.

Attenuated Total Reflectance (ATR) Fourier-Transform Infrared (ATR-FTIR) spectra were recorded on a Spectrum One FTIR spectrometer of PerkinElmer Instruments equipped with a Universal ATR Sampling Accessory (Waltham, MA, USA). The polymer was irradiated by IR beam through a special diamond-zinc-selenium composite prism. The penetration depth of the IR beam in the material is at about 6 μm. The average film thickness of the specimens was ca. 0.5 mm. Four scans were taken for each sample. The spectra were evaluated by Spectrum ES 5.0 program (PerkinElmer, Waltham, MA, USA).

Instron 4302 type testing machine (Buckinghamsire, UK), equipped with a 1 kN load cell, was used for tensile testing of the EP-PU series. At least three dumbbell specimens were cut (clamped length 60 mm) from the film samples and tensile loaded at a strain rate (dε/dt) of 0.025/s. 

The thermal properties of the synthesized PUs were examined by Differential Scanning Calorimetry (DSC). DSC tests were carried out in a DSC Q2000 power compensation equipment (TA Instruments, Hüllhorst, Germany) operating at 10 °C/min heating rate. Nitrogen was used as protective atmosphere. The weight percentage of the crystalline PCL (C_r_ (%)) was calculated by Equation (3) [25]:

(3) $$C_{r}(\%)=\frac{\Delta H_{m}}{w_{A}\Delta H_{m}^{o}}\cdot 100$$

where Δ*H*_m_ is the heat of fusion of the PCL-containing PU, *w*_A_ is the weight fraction of PCL in the corresponding PU, and $\Delta H_{m}^{o}$ is the heat of fusion of the pure 100% crystalline PCL. For the latter, 135.31 J/g has been taken from [26].

Dynamic Mechanical Analysis (DMA) testing of the PUs was carried out in a DMA Q800 device (TA Instruments, Hüllhorst, Germany). DMA traces were monitored in tension mode (dimension of the specimens: length: 25 mm, clamped length: 12 mm, width: 7 mm, thickness: ca. 0.5 mm) at an oscillation amplitude of 0.2% setting a frequency of 1 Hz and a static load of 1 N. The temperature was varied between −50 and 200 °C with a heating rate of 3 °C/min.

Shape memory properties were determined in tensile mode using the aforementioned DMA device. The specimens (ca. 12 × 7 × 0.5 mm) were stretched after 10 min holding at 60 °C at a strain rate of 15 %/min to 30% strain, followed by a quick cooling to 20 °C (cooling rate: 4.44 °C/min) and the shape fixity (*R*_f_) was determined in 1 min after the stress had been released. Shape recovery (*R*_r_) was measured at 0.1 N loading of the specimens (quasi free recovery) by reheating the specimens at 3 °C/min heating rate from 20 to 60 °C and holding there for 20 min. The shape fixity (*R*_f_) and shape recovery ratios (*R*_r_) are determined according to Equations (4) and (5):

(4) $$R_{f}(\%)=\frac{l_{d}-l_{o}}{l_{30\%}-l_{o}}\cdot 100$$

(5) $$R_{r}(\%)=\frac{l_{d}-l_{f}}{l_{d}-l_{o}}\cdot 100$$

where *l*_d_ is the sample length after removal of the tensile load during shape fixing at 20 °C, l_o_ is the clamped length of the sample at 20 °C, *l*_30%_ is the length after stretching the sample at 60 °C in tensile load, and *l*_f_ is the final recovered length of the stretched specimen.

To get a deeper insight into the morphology Scanning Electron Microscopic (SEM) was adopted. SEM pictures were taken from the surface of selected specimens by a Hitachi S-4800 equipment (Tokyo, Japan). Surface of the specimens was covered with a 30 nm conductive gold layer.

## 3. Results and Discussion

In the syntheses of EP-PU 1–24, the *M*_n_ of PCL, the amounts of DGEBA or FA_DGEBA oligomer, BMI and the HO-ended DA adduct were systematically varied to study their effects on the properties and morphologies of the resulting EP-PUs. Recall that two synthetic approaches were followed to prepare EP-PU co-networks. In the case of sample EP-PU 1–15 (see Table 1), the HO-ended DA adduct was incorporated as a thermoreversible connecting element between the isocyanate terminated polyurethane prepolymer and DGEBA, as depicted in Scheme 1. Furthermore, in the case of some EP-PU systems, crosslinking of EP was achieved using Jeffamine (JA) (EP-PU 10–15). In the second approach (see Table 2), the DA-adduct was obtained “in situ” through the reaction between the furanyl- and maleimide-containing prepolymers, as shown in Scheme 2. The latter was obtained by the reaction of isocyanate functionalized prepolymer with FA (see Figure 2). To link the FA-end-functionalized prepolymers to the FA_DGEBA oligomer, BMI was added to the reaction mixture, which led to formation of the DA-adducts (see EP-PU 16–24). It is noteworthy that only selected EP-PU samples were subjected to some investigations to keep this contribution in limits. However, peculiar attention was paid to their selection to obtain information on effects of relevant compositional/structural variables. 

### 3.1. Infrared Spectroscopy of the EP-Networks

**Figure 3:**
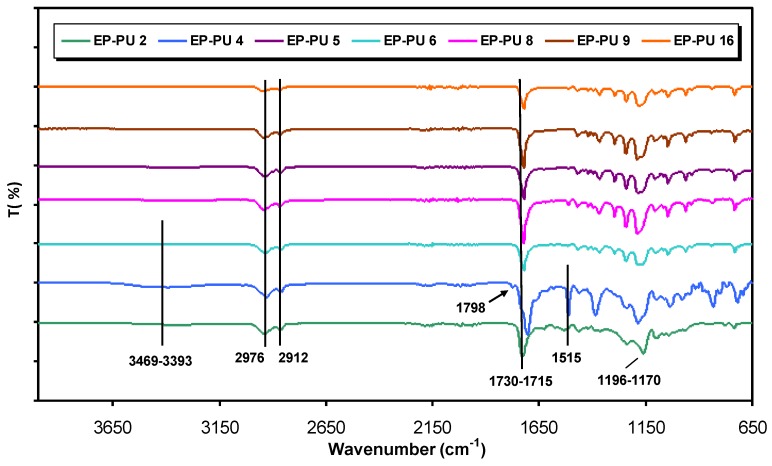
ATR-FTIR spectra of EP-PUs 2, 4, 5, 6, 8, 9 and 16.

Figure 3 shows the ATR-FTIR spectra of the representative samples EP-PU 2, 4, 5, 6, 8, 9 and 16.

In the case of EP-PU samples with PCL of lower molecular weight (e.g., EP-PU 2 and 4), the NH band of urethane can be detected at 3393–3469 cm^−1^ as a broad absorption. The absorption bands at 2912 and 2976 cm^−1^ and at 1362 cm^−1^ belong to the symmetric and asymmetric CH_2_ stretching and bending vibrations, respectively. The lack of absorption band at around 2230 cm^−1^, which is characteristics of the NCO group, indicates the complete transformation of the NCO end groups. The C=O band of EP-PUs is visible at 1715–1730 cm^−1^, which suggests that there is no considerable hydrogen bond interaction. In the FTIR spectra of PUs, the H-bonded C=O band should appear at 1684 and 1690 cm^−1^ [27].

The weak absorption band at 1798 cm^−1^ (C=O of maleimide part) appeared only in the FTIR spectrum of EP-PU 4 indicating the presence of the DA adduct [23]. Moreover, in the case of EP-PU samples prepared with higher molecular weight PCLs (e.g., 10, 25 and 50 kg/mol), this band is not visible. Another intriguing finding is the presence of a band at 1515 cm^−1^, which may be associated with the amide II band for poly(ester-ether urethane) [28]. This band occurred with much higher intensity for the sample EP-PU 4 than for other PU samples. The band at ~1196–1170 cm^−1^ with relatively high intensity was assigned to the =C–O/–C–O–C– vibrations which appeared as a sharp peak in the FTIR spectrum of EP-PU 2 [29].

### 3.2. Thermal Behavior

**Figure 4:**
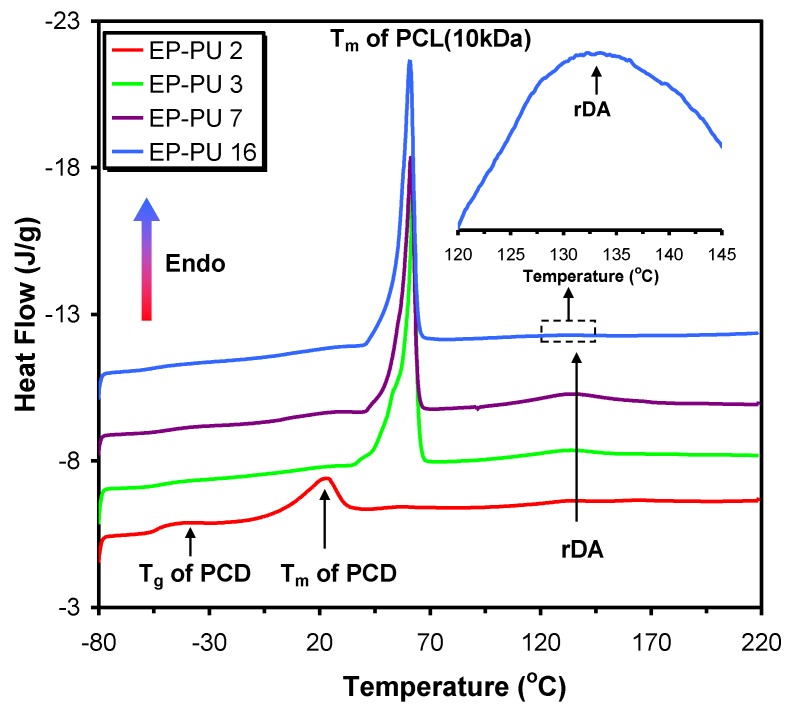
DSC curves of EP-PUs 2, 3, 7 and 16. The inset shows the zoomed part of the DSC trace for the sample EP-PU 16 in the temperature range of 120–145 °C.

The thermal behaviors of the EP-PU samples were investigated by DSC. Some typical DSC traces for the EP-PU samples are shown in Figure 4 and the related results listed in Table 3.

According to Figure 4 and the data in Table 3, the glass transition temperature (*T*_g_) of the samples containing PCD with *M*_n_ = 2 kg/mol appears in the range from −50 to −40 °C, while *T*_m_ values are around 20 °C (see Table 3). However, T_m_-s of higher molecular weight PCL segments (e.g., *M*_n_ = 10 kg/mol) appear at around 60 °C, which is in good agreement with former works on PUs with PCL segments [30,31]. In the case of samples with increasing EP contents (see EP-PU 2 and 6), the Δ*H*_m_, and thus the crystallinity values, decreased considerably. Moreover, the degree of crystallinity also decreased with crosslinking of EP. This can be traced to the fact that crosslinking reduces the flexibility of PCL segments and thus decreases the crystallizable fraction of PCL in these samples (see EP-PU 3 vs. EP-PU 10 and EP-PU 7 vs. EP-PU 13 and EP-PU 4 vs. EP-PU 11). An important result of the DSC tests was that, besides the T_g_ and T_m_, a broad endothermic peak could be resolved in DSC traces in the temperature range 110–145 °C (see EP-PU 2, 3, 7 and 16). This was assigned to the retro-DA (rDA) reaction.

### 3.3. Morphology

**Figure 5:**
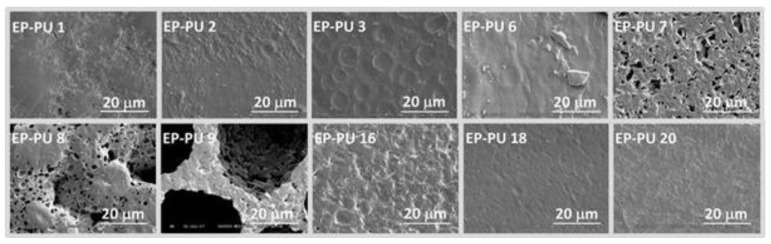
SEM images of samples EP-PU 1, 2, 3, 6, 7, 8, 9, 16, 18 and 20.

As seen in Figure 5 the morphology considerably changed by the synthetic method and chemical composition applied for the preparation of these samples. For example, the morphology of samples EP-PU 2, 3, 6, 16, 18 and 20 seems to be more uniform, than those of EP-PUs 7, 8 and 9. Furthermore, with increasing amount of the EP component, a “voided” structure was formed, which might be caused by a strong microphase separation in the co-networks. This structural feature becomes more pronounced with increasing molecular weight of PCL segments (see, e.g., EP-PUs 6–9).

### 3.4. Tensile Properties

The results of tensile tests of the samples prepared by the two synthetic approaches are presented in Table 4 and Table 5. 

The data in Table 4 indicate that EP-PU samples prepared with the low molecular weight PCD (2 kg/mol) exhibit low E-moduli and relatively high elongation at break values (see EP-PU 2 and 6). Furthermore, it can be noticed that the M_n_ of the PCL also greatly affected the mechanical properties, especially the E-modulus. Samples with the highest E-moduli were obtained with PCL of *M*_n_ = 25 and 50 kg/mol, respectively (see samples EP-PU 4 and EP-PU 5). Interestingly, incorporation of higher amount of DA adducts into the EP-PU system yielded lower stiffness (E-modulus), tensile strength and elongation at break values (compare e.g., EP-PU 2 with EP-PU 6, EP-PU 3 with EP-PU 7, EP-PU 4 with EP-PU 8). This tendency can be attributed to the fact that, in the presence of higher amount of DA-adducts, shorter –DA-PU– chains were formed. It has to be underlined that crosslinking trough the remaining epoxy groups with a polyether amine (Jeffamine^®^ EDR 176) yielded co-networks whose E-moduli and tensile strength were higher than those of their non-crosslinked counterparts (compare EP-PU 7 with EP-PU 13 and EP-PU 4 with EP-PU 11).

Although the mechanical properties of the EP-PU systems prepared by the second synthetic approach (i.e., containing polymerized EP resin and DA adducts) do not show clear trends, some important conclusions can be drawn from the data in Table 5: (i) at low EP resin content, the increasing amount of the DA coupling agent considerably reduced the elongation at break (see samples EP-PU 16 and EP-PU 17); (ii) increase in the amount of EP resin in the samples prepared with PCL of *M*_n_ = 10 kg/mol resulted in higher E-moduli (see samples EP-PU 16–20), while an opposite trend can be observed for the samples with PCL of *M*_n_ = 50 kg/mol (see samples EP-PU 21 to 24); (iii) the elongation at break decreased with increasing EP content and with the M_n_ of PCL; and (iv) the E-moduli are higher and the elongations at break values for the EP-PUs prepared by the second synthetic route (Scheme 2) are lower than those prepared by the first one (Scheme 1). 

The stress–strain curves for samples prepared with low molecular weight PCD (*M*_n_ = 2 kg/mol) and PCL (*M*_n_ = 10 kg/mol) resembled to those of elastomers while samples made with PCL of higher *M*_n_ or prepared by the second synthetic approach showed tensile characteristics similar to those of flexible plastics.

### 3.5. Modeling the Stress–Strain Behavior the EP-PU Co-networks

**Figure 6:**
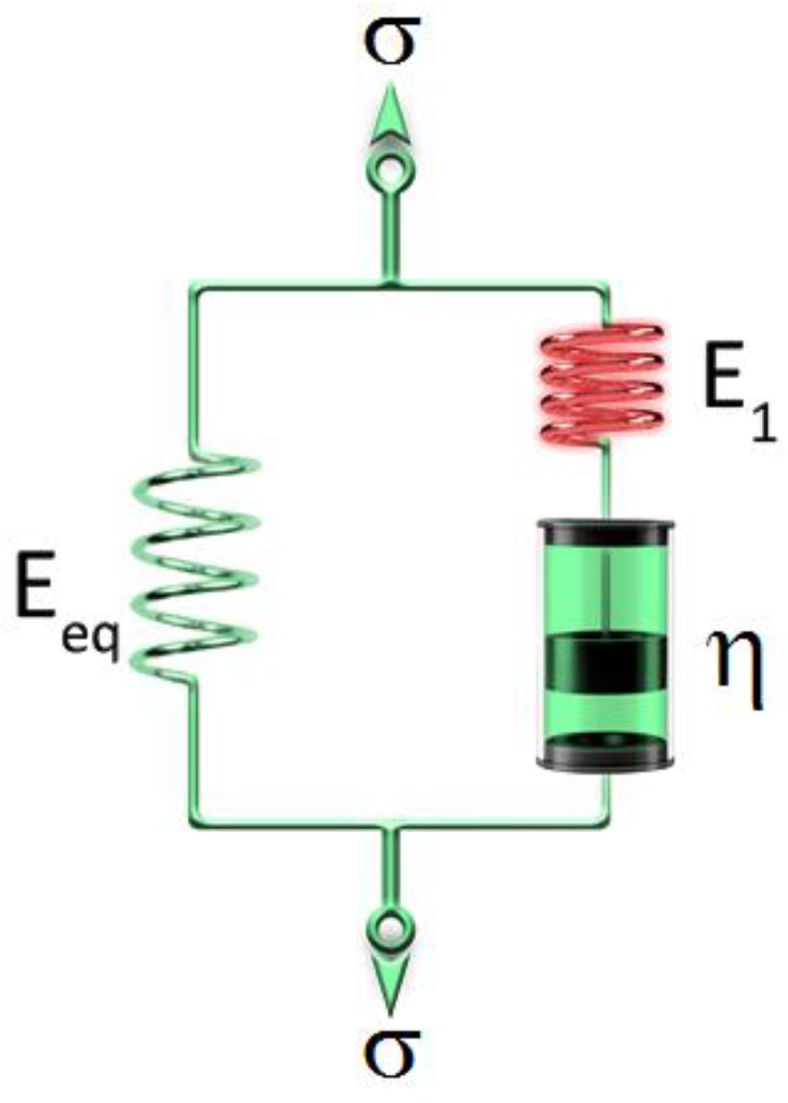
The Standard Linear Solid (SLS) model. *E*_eq_ and E_1_ represent the equilibrium (rubbery) and the non-equilibrium (glassy) spring modulus, respectively, while η stands for the dynamic viscosity of the liquid in the dashpot.

In the following, it will be shown that the observed stress–strain curves for some synthesized EP-PU samples having more uniform micromorphologies (EP-PU 1 and 2) can be adequately described using the Standard Linear Solid (SLS) rheological model presented in Figure 6.

According to this model, the relationship between the stress (σ) and the strain (ε) as well as their derivatives with respect to time are given by Equation (6) [32]

(6) $$\sigma +a_{1}\frac{d\sigma }{dt}=a_{2}\varepsilon +a_{3}\frac{d\varepsilon }{dt}$$

where *a*_1_ = η/*E*_1_; *a*_2_ = *E*_eq_; and *a*_3_ = η(1 + *E*_eq_/*E*_1_)

In uniaxial tensile experiments, the strain rate (*dε*/*dt*) was kept constant, thus Equation (6) can be rewritten to obtain Equation (7):

(7) $$\sigma +a_{1}\left(\frac{d\varepsilon }{dt}\right)\frac{d\sigma }{d\varepsilon }=a_{2}\varepsilon +a_{3}\frac{d\varepsilon }{dt}$$

Integrating Equation (7) yields Equation (8), which establishes a relationship between the stress (*σ*) and strain (*ε*) at a constant strain rate.

(8) $$\sigma =b_{1}\left[\varepsilon +b_{2}(1-e^{-b_{3}\varepsilon })\right]$$

where $b_{1}=a_{2}$, $b_{2}=(d\varepsilon /dt)(a_{3}/a_{2}-a_{1})$ and $b_{3}=\frac{1}{(d\varepsilon /dt)a_{1}}$.

**Figure 7:**
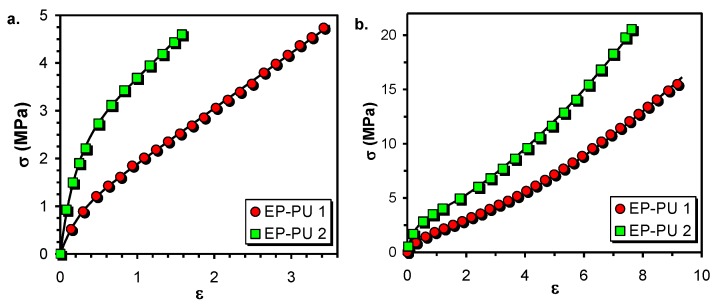
Stress–strain curves for the samples EP-PUs 1 and 2: up to moderate strains (**a**); and up to elongation at break (**b**). The symbols and the solid lines represent the experimental data and the fitted curves, respectively. The fitted parameters for the sample EP-PU 1 are *b*_1_ = 1.16 MPa, *b*_2_ = 0.63 and *b*_3_ = 4.73 (i.e., (*dε*/*dt*)*a*_1_ = 0.21, *a*_2_ = 1.16 MPa, (*dε*/*dt*)*a*_3_ = 0.97 MPa and α = 0.089 MPa), while for the sample EP-PU 2 these parameters are *b*_1_ = 1.57 MPa, *b*_2_ = 1.36 and *b*_3_ = 5.17 (i.e., *dε*/*dt*)*a*_1_ = 0.19, *a*_2_ = 1.57 MPa, (*dε*/*dt*)*a*_3_ = 2.44 MPa and α = 0.15 MPa).

Parameters *b*_1_, *b*_2_ and *b*_3_ were obtained by fitting Equation (8) to the corresponding experimental σ–ε data points. As evident from Figure 7a, Equation (7) describes the stress–strain curves accurately up to moderate strain values.

On the other hand, it can also be concluded from Figure 7a and the fitted parameters that increasing the molar ratio of the Diels–Alder adduct from 0.5:0.5 to 1:1 (compare EP-PU 1 with EP-PU 2) results in the increase of parameters b_1_, b_2_ and b_3_. This indicates an increase in the E-modulus and a steeper dependence of *σ* on *ε*. This finding can be ascribed to a higher crosslink density in the case of EP-PU 2. As demonstrated above, the relatively simple SLS model describes accurately the stress–strain relationship up to moderately high strains, however, it fails at high strains due to the considerable upward curvature of the *σ* vs. *ε* traces. Because the latter feature should be linked with strain-induced crystallization, it has to be considered to describe the *σ–ε* curves at high strains. Therefore, it was assumed that, after a certain strain (*ε_L_*), strain-induced orientation/crystallization takes place, which manifests in an increased value of *E*_eq_, i.e., in the value of a_2_(*ε*) parameter, as shown by Equation (9a) and Equation (9b).

(9a) $$a_{2}(\varepsilon )=a_{2}\;(\mathrm{if}\;\varepsilon \leq \varepsilon_{L})$$

(9b) $$a_{2}(\varepsilon )=a_{2}+\alpha (\varepsilon -\varepsilon_{L})\;(\mathrm{if}\;\varepsilon >\varepsilon_{L})$$

where *a*_2_ is the value of *a*_2_(*ε*) up to the onset of curvature of the *σ–ε* curve and α is a proportionality constant.

After an appropriate rearrangement of Equation (6) and using Equation (9b), we get the following equation:

(10) $$\frac{d\sigma }{d\varepsilon }=\frac{1}{a_{1}\frac{d\varepsilon }{dt}}(a_{2}(\varepsilon )\varepsilon +a_{3}\frac{d\varepsilon }{dt}-\sigma )$$

Equation (10) was integrated numerically and fitted to the whole *σ–ε* curves (up to break) to obtain parameter α. It should be noted, however, that, for fitting to the whole curve, parameters a_1_, a_2_ and a_3_ were used as determined from the *σ–ε* curves up to moderately strains and only parameter α was that one which was obtained by fitting of Equation (10).

As seen in Figure 7b, the extended SLS model (Equation (10)) is capable of describing the *σ* vs. *ε* relationship for the EP-PU systems in a wide range of *ε* values.

### 3.6. DMA Analysis

**Figure 8:**
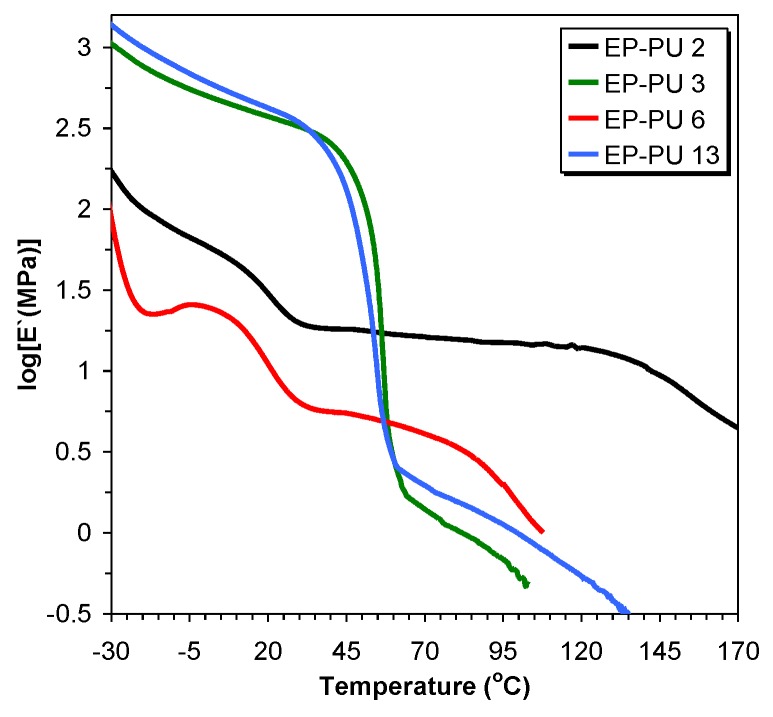
Storage modulus as a function of temperature for samples EP-PU 2, 3, 6, and 13.

Thermomechanical behavior of samples EP-PUs was also studied using dynamic mechanical analysis (DMA) in tensile mode. Storage modulus as a function of temperature for samples EP-PU 2, 3, 6 and EP-PU 13 are depicted in Figure 8. The crosslink densities (*ν*_e_) were calculated by Equation (11) and are summarized in Table 6.

(11) $$\nu_{e}=\frac{E^{\prime }}{3RT}$$

where *E*′, *R* and *T* are the storage modulus, the universal gas-constant and the temperature at the onset of the rubbery state, respectively. Since no clear rubbery plateau appeared, the *E*′ was read at the onset temperature of the rubbery state (cf. Table 6).

As shown in Figure 8., the storage modulus decreased (*E*′) considerably at 20–22 °C for EP-PU 2 and EP-PU 6, while for the sample EP-PU 3 (containing crystalline PCL) E′ decreased sharply only at 60–65 °C due to the melting of the crystalline PCL phase. It is noteworthy that well developed rubbery plateau, attributable to the presence of a crosslinked network, was rarely observed (e.g., EP-PU 2). Instead, the *E*′ was reduced with increasing temperature featuring a leathery transition. The width of the latter decreased with increasing molar ratio of EP to PU (compare the DMA curve of EP-PU 2 and EP-PU 6). The *E*′ decrease in the rubbery plateau/leathery region reflects most likely the advancing rDA reaction. The rDA process lowers the crosslink density and/or the segments lengths between the netpoints, thus yielding lower E′ values. 

### 3.7. Shape Memory Experiments

The SM characteristics, i.e., the values of shape fixity (*R*_f_) and shape recovery ratios (*R*_r_), are given in Table 7. As shown in the data in Table 7, the shape fixity ratios are very close to 100% for samples EP-PU3, EP-PU 10 and EP-PU13. The high values of *R*_f_ can be traced to the significant difference in the “glassy” and the “rubbery” moduli [32,33,34]. However, poor shape fixity ratios were found for EP-PU 2 most likely due to the low melting temperature of the PCD segments (cf. Table 3). On the other hand, decreasing the shape fixity temperature from 20 to 0 °C for sample EP-PU 2 results in a better R_f_ value. The related original DMA traces registered during SM programing of EP-PU 2, i.e., temperature, stress and strain as a function of time, are given in the Supplementary Materials (see Figures S1 and S2, respectively). In the Supplementary Materials, we have also included the original SM cycles for EP-PU 3 (Figure S3) and EP-PU 10 (Figure S4) to demonstrate effects of the additional crosslinking with Jeffamine (JA, see Table 1). Note that, except for EP-PU 2, rather high *R*_r_ values were measured for all other tested samples.

Since the recovery process plays an important role in the function of shape memory polymers, an attempt was made to describe the relationship between the strain (ε) and the recovery time (t). It was found that the strain as a function of time can be fairly described by means of a double exponential decay function as shown by Equation (12). Note that this approach was suited to describe the strain recovery also for another PU system [34]:

(12) $$\varepsilon (t)=A_{1}e^{\frac{-(t-t_{o})}{\tau_{1}}}+A_{2}e^{\frac{-(t-t_{o})}{\tau_{2}}}$$

where *ε*(*t*) is the strain, *A*_1_ and *A*_2_ are amplitudes (pre-exponential factors), τ_1_ and τ_2_ represent relaxation times, and *t*_o_ is the onset of shape recovery (accordingly, *t* ≤ *t*_o_ is the valid range).

**Figure 9:**
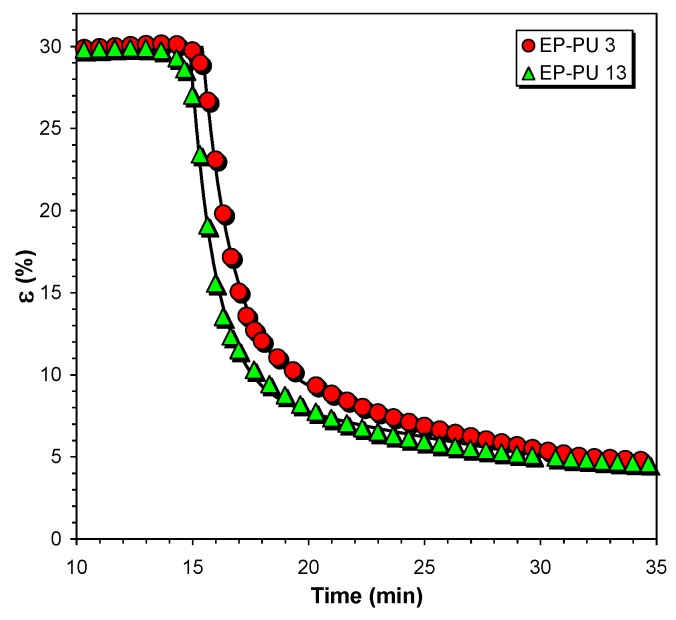
Free recovery curves for the EP-PU 3 and EP-PU 13 samples. The symbols and solid lines represent the experimental data and the fitted curves calculated using double exponential functions, respectively. The fitting parameters of the curves are: *A*_1_ = 18.6, τ_1_ = 1.23 min^−1^ and *A*_2_ = 11.0, τ_2_ = 21.2 min^−1^ for the EP-PU 3, *A*_1_ = 17.1, *τ*_1_ = 1.1 min^−1^ and *A*_2_ = 9.2, τ_2_ = 25.0 min^−1^ for the EP-PU 13.

In Figure 9, the close isothermal free recovery as a function of time for the samples EP-PU 3 and 13 are plotted.

As confirmed in Figure 9, Equation (12) describes the variation of strain with time adequately. The first, relatively fast, relaxation (τ_1_) can be ascribed to the strain release due to the rapid melting of the crystalline PCL segment, while the second, much slower, relaxation process (τ_2_) is most likely linked with the time required for polymer chains to return to their original conformations [34].

## 4. Conclusions

EP-PU co-networks containing PCL and DA adducts were prepared using two different synthetic strategies. The first synthetic way for the preparation of samples EP-PU 1–15 was the incorporation of a HO-ended DA adduct in the networks composed of isocyanate (HDI) terminated polyurethane prepolymer and DGEBA and followed by crosslinking of the EP-component by polyetheramine (Jeffamine^®^ EDR 176). The other synthetic route utilized the reaction between furanyl end-functionalized PU polymers and FA_DGEBA oligomer in the presence of BMI as the coupling agent (samples EP-PU 16–24). Thus, the two synthetic routes differed from one another in generation of the DA-adducts and in the composition of the co-networks. The morphology of the EP-PU samples, investigated by SEM, also varied with the composition and the synthetic method. The presence of the DA adducts in the EP-PU samples synthesized was supported by ATR-FTIR and DSC measurements. According to the results of DMA, above *T*_m_ of the PCL, a rubbery plateau or a leathery region was obtained for the EP-PU samples. It was proposed that the incorporated DA-adducts provide an additional switch via rDA reactions in addition to the crystalline PCL segments, carrying a possibility for additional shape memory programming. The stress–strain curves of the EP-PU samples could be well approximated by the simple SLS model up to moderately low strains. By incorporating a strain-induced crystallization term, the validity range of the SLS model could be expanded for high strains, as well. It has also been shown that the free recovery process in shape memory tests of the EP-PU samples can adequately be described by double exponential decay functions including fast and slow relaxation modes.

These novel EP-PU co-networked systems containing reversible DA-linkages may find their applications among the well-tuned T_m_-based shape memory and self-healing polymers.

## Figures and Tables

**Table:** 

Compound	Protons	σ(ppm)/CDCl_3_
FA	A	7.32
	B	6.30
	C	6.11
	D	3.79
	NH_2_	1.75
DGEBA	ArH	7.11 and 6.80
	1 (CH_2_ epoxy ring)	2.87, 2.71
	2 (CH epoxy ring)	3.32
	3 (-CH_2_-O-)	4.16, 3.92
	-CH_3_	1.61
FA_DGEBA-oligomer	a	7.25
	b	6.80
	c	6.19
	d	3.81
	1′ (CH_2_ from epoxy ring)	2.85–2.75
	2′ (CH from epoxy ring)	4.06
	3′ (-CH_2_-O-)	3.81, 3.92
	-OH	4.06
	-CH_3_	1.61

**Table 1:** The corresponding molar ratios in the feed of the EP-PUs 1–15 synthesized. The number in the parenthesis represents the *M_n_* of the PCD or PCL expressed in kg/mol unit.

Sample	Composition/Molar Ratios in the Feed
EP-PU 1	PCD(2)-HDI-DA adduct-DGEBA/1:2:0.5:0.5
EP-PU 2	PCD(2)-HDI-DA adduct-DGEBA/1:2:1:1
EP-PU 3	PCL(10)-HDI-DA adduct-DGEBA/1:2:1:1
EP-PU 4	PCL(25)-HDI-DA adduct-DGEBA/1:2:1:1
EP-PU 5	PCL(50)-HDI-DA adduct-DGEBA/1:2:1:1
EP-PU 6	PCD(2)-HDI-DA adduct-DGEBA/1:2:2:2
EP-PU 7	PCL(10)-HDI-DA adduct-DGEBA/1:2:2:2
EP-PU 8	PCL(25)-HDI-DA adduct-DGEBA/1:2:2:2
EP-PU 9	PCL(50)-HDI-DA adduct-DGEBA/1:2:2:2
EP-PU 10	PCL(10)-HDI-DA adduct-DGEBA-JA/1:2:1:1:1
EP-PU 11	PCL(25)-HDI-DA adduct-DGEBA-JA/1:2:1:1:1
EP-PU 12	PCL(50)-HDI-DA adduct-DGEBA-JA/1:2:1:1:1
EP-PU 13	PCL(10)-HDI-DA adduct-DGEBA-JA/1:2:2:2:2
EP-PU 14	PCL(25)-HDI-DA adduct-DGEBA-JA/1:2:2:2:2
EP-PU 15	PCL(50)-HDI-DA adduct-DGEBA-JA/1:2:2:2:2

**Table 2:** Composition and the corresponding molar ratios in the feed of the EP-PUs 16–24 synthesized. The number in the parenthesis represents the M_n_ of the PCL expressed in kg/mol unit.

Sample	Composition/Molar Ratios in the Feed
EP-PU 16	PCL(10)-HDI-FA-FA_DGEBA oligomer-BMI/1:2:2:1:1
EP-PU 17	PCL(10)-HDI-FA-FA_DGEBA oligomer-BMI/1:2:2:1:2
EP-PU 18	PCL(10)-HDI-FA-FA_DGEBA oligomer-BMI/1:2:2:2:1
EP-PU 19	PCL(10)-HDI-FA-FA_DGEBA oligomer-BMI/1:2:2:2.5:1
EP-PU 20	PCL(10)-HDI-FA-FA_DGEBA oligomer-BMI/1:2:2:3:1
EP-PU 21	PCL(50)-HDI-FA-FA_DGEBA oligomer-BMI/1:2:2:5:1
EP-PU 22	PCL(50)-HDI-FA-FA_DGEBA oligomer-BMI/1:2:2:10:1
EP-PU 23	PCL(50)-HDI-FA-FA_DGEBA oligomer-BMI/1:2:2:12.5:1
EP-PU 24	PCL(50)-HDI-FA-FA_DGEBA oligomer-BMI/1:2:2:15:1

**Table 3:** Glass transition temperature (*T*_g_), melting temperature (*T*_m_), melting enthalpy (Δ*H*_m_), crystallinity values and rDA temperatures from DSC measurements in the first heating cycle.

Sample	*T*_g_ (°C)	*T*_m_ (°C)	Δ*H*_m_ (J/g)	Crystallinity (%)	rDA (°C)
EP-PU 1	−48.4	23.8	9.8	10.1	133.9
EP-PU 2	−53.1	22.9	19.4	23.3	136.8
EP-PU 3	-	61.7	67.8	56.3	134.6
EP-PU 4	-	65.3	89.95	69.7	139.8
EP-PU 6	−41.7	20.3	3.7	5.7	138.4
EP-PU 7	-	61.3	67.6	60.7	135.3
EP-PU 10	-	61.8	57.8	48.4	133.3
EP-PU 11	-	63.2	73.1	56.9	136.4
EP-PU 13	-	61.6	54.9	50.0	132.7
EP-PU 16	-	60.9	64.2	58.6	133.7
EP-PU 17	-	62.8	67.4	61.5	131.1
EP-PU 18	-	62.2	67.0	66.5	130.8
EP-PU 20	-	61.3	58.6	64.6	128.9

**Table 4:** Tensile mechanical properties of the EP-PUs 1–15. Designations: *E*, elastic modulus; ε_R_, ultimate elongation; σ_R_, stress at break.

Sample	*E* (MPa)	ε_R_ (%)	σ_R_ (MPa)
EP-PU 1	2 ± 0.2	837 ± 26	17 ± 2
EP-PU 2	4 ± 1	627.5 ± 77	20 ± 2
EP-PU 3	177 ± 14	974 ± 76	27 ± 3
EP-PU 4	233 ± 5	4 ± 0.2	7.5 ± 0.5
EP-PU 5	181 ± 12	794.5 ± 50	20 ± 3
EP-PU 6	1 ± 0.2	910 ± 31	9 ± 1
EP-PU 7	107 ± 10	30 ± 5	7 ± 0.2
EP-PU 8	184 ± 13	5 ± 1	3 ± 1
EP-PU 9	92 ± 16	298 ± 30	8.2 ± 0.5
EP-PU 10	232 ± 5	851 ± 119	25 ± 1
EP-PU 11	237 ± 29	457 ± 63	17 ± 1
EP-PU 12	349 ± 20	907.5 ± 6	35 ± 0.3
EP-PU 13	221 ± 29	604.5 ± 31	22.5 ± 0.7
EP-PU 14	124.5 ± 16	243.5 ± 35	10 ± 0.7
EP-PU 15	208 ± 19	549 ± 65	21 ± 1

**Table 5:** Tensile mechanical properties of EP-PUs 16–24. For designations, see Table 4.

Sample	*E* (MPa)	ε_R_ (%)	σ_R_ (MPa)
EP-PU 16	387 ± 10	670 ± 113	22 ± 3
EP-PU 17	388 ± 98	378 ± 83	21 ± 1
EP-PU 18	392 ± 6	359 ± 8	14 ± 3
EP-PU 19	470 ± 107	2 ± 0.3	10 ± 2
EP-PU 20	648.5 ± 47	3.5 ± 0.5	15 ± 2
EP-PU 21	477 ± 51.5	419 ± 42	23 ± 1
EP-PU 22	366 ± 31	11 ± 2	13 ± 0.3
EP-PU 23	294 ± 66	9 ± 1.4	10 ± 1
EP-PU 24	305 ± 24	11.5 ± 3	11 ± 0.3

**Table 6:** The *E*′ values and crosslink densities (*ν*_e_) obtained from DMA results for samples EP-PU 2, 3, 6 and EP-PU 13. The temperature in the bracket after the value of *ν*_e_ is the rubbery onset temperature considered in the calculation.

Sample	*E*′ (MPa)	*ν*_e_ (moL/cm^3^)
EP-PU 2	18.2	2.3 × 10^−3^ (40 °C)
EP-PU 3	1.5	1.8 × 10^−4^ (67 °C)
EP-PU 6	5.6	7.2 × 10^−4^ (40 °C)
EP-PU 13	2.2	2.6 × 10^−4^ (65 °C)

**Table 7:** Shape fixity and shape recovery ratios for selected EP-PUs.

Sample	*R*_f_ (%)	*R*_r_ (%)
EP-PU 2	35	77
	63 *	88 *
EP-PU 3	99	83
EP-PU 10	99	84
EP-PU 13	98	85

* Shape fixed at 0 °C.

## Supplementary Materials

Supplementary materials are available online at http://www.mdpi.com/2073-4360/10/5/504/s1.
