# Supplementary material for: One-Pot Synthesis and Characterization of Novel Shape-Memory Poly(ε-Caprolactone) Based Polyurethane-Epoxy Co-networks with Diels–Alder Couplings
Source: Polymers (Basel). 2018 May 6;10(5):504. doi: 10.3390/polym10050504 (PMC6415404; doi:10.3390/polym10050504)
Supplement: Supplementary file 1 [file polymers-10-00504-s001.doc]

**One-pot synthesis and characterization of novel shape-memory poly(ε-caprolactone) based polyurethane-epoxy conetworks with Diels-Alder couplings**

Katalin Czifrák^1^, Csilla Lakatos^1^, József Karger-Kocsis^2^, Lajos Daróczi^3^, Miklós Zsuga^1^, Sándor Kéki^1,*^

*^1^ Department of Applied Chemistry, University of Debrecen, Egyetem tér1, H-4032 Debrecen, Hungary*

*^2^ Department of Polymer Engineering, Budapest University of Technology and Economics, Műegyetem rkp. 3, H-1111 Budapest, Hungary*

*^3^ Department of Solid State Physics, University of Debrecen, Bem tér 18/b, H-4026 Debrecen, Hungary*

**Table of contents**

**Figure S1.** Shape memory cycle of EP-PU 2 with 30 % strain at 20 °C shape fixity temperature

**Figure S2.** Shape memory cycle of EP-PU 2 with 30 % strain at 0 °C shape fixity temperature

**Figure S3.** Shape memory cycle of EP-PU 3 with 30 % strain at 20 °C shape fixity temperature

**Figure S4.** Shape memory cycle of EP-PU 10 with 30 % strain at 20 °C shape fixity temperature

**Figure S1.** Shape memory cycle of EP-PU 2 with 30 % strain at 20 °C shape fixity temperature

**Figure S2.** Shape memory cycle of EP-PU 2 with 30 % strain at 0 °C shape fixity temperature


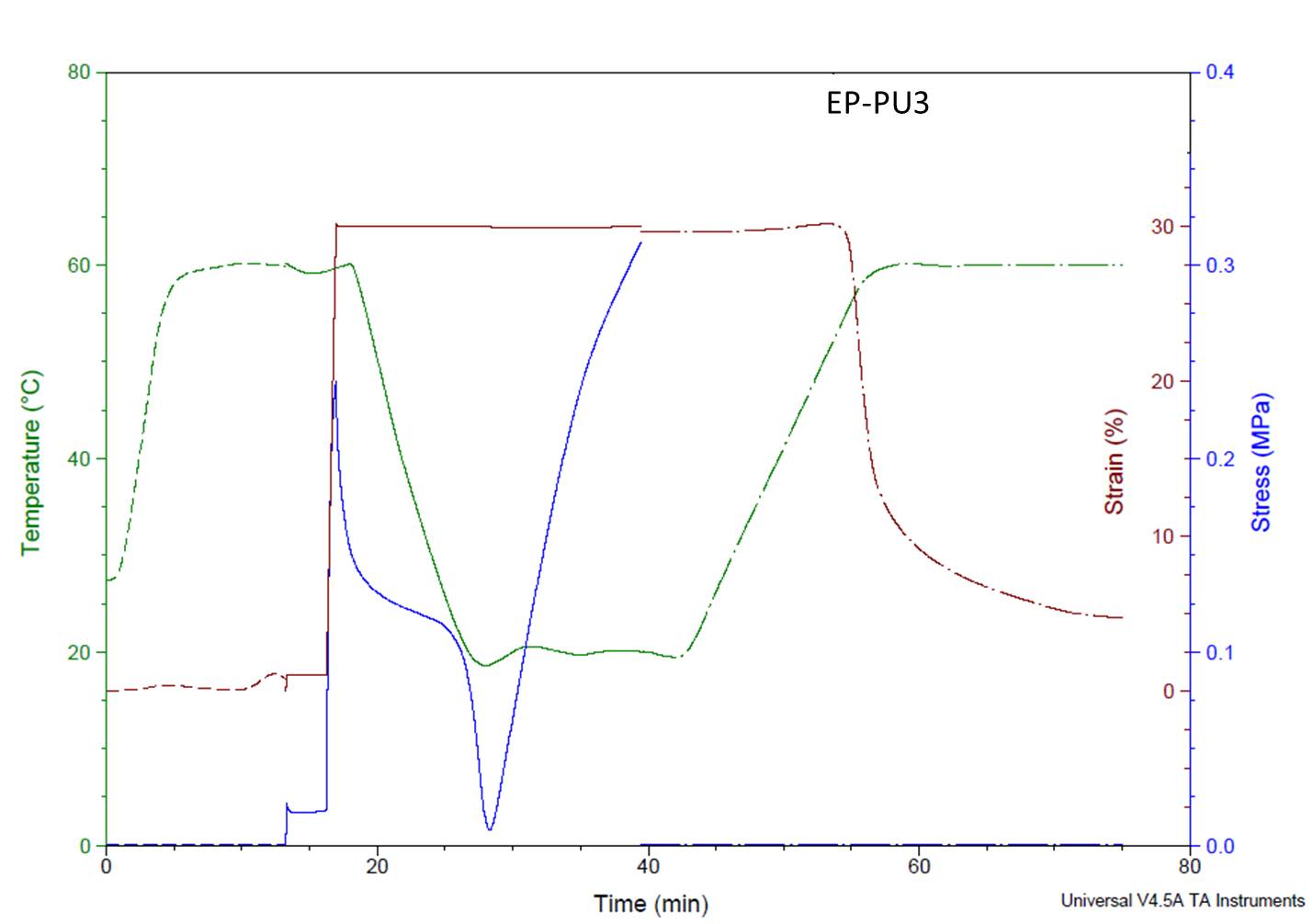


**Figure S3.** Shape memory cycle of EP-PU 3 with 30 % strain at 20 °C shape fixity temperature


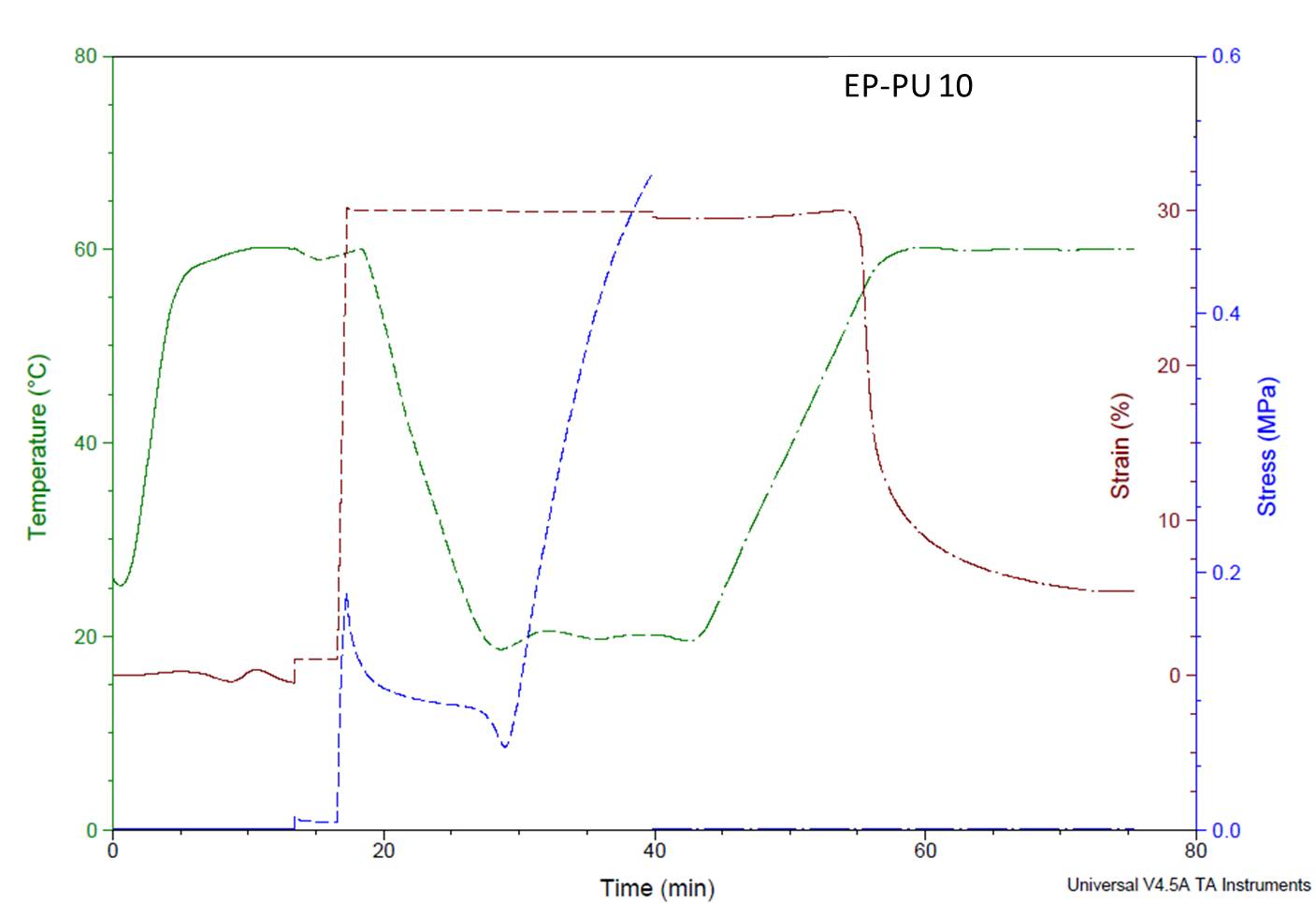


**Figure S4.** Shape memory cycle of EP-PU 10 with 30 % strain at 20 °C shape fixity temperature
